# MRI texture features from tumor core and margin in the prediction of response to neoadjuvant chemotherapy in patients with locally advanced breast cancer

**DOI:** 10.18632/oncotarget.28002

**Published:** 2021-07-06

**Authors:** Christopher Kolios, Lakshmanan Sannachi, Archya Dasgupta, Harini Suraweera, Daniel DiCenzo, Gregory Stanisz, Arjun Sahgal, Frances Wright, Nicole Look-Hong, Belinda Curpen, Ali Sadeghi-Naini, Maureen Trudeau, Sonal Gandhi, Michael C. Kolios, Gregory J. Czarnota

**Affiliations:** ^1^Physical Sciences, Sunnybrook Research Institute, Sunnybrook Health Sciences Centre, Toronto, Canada; ^2^Department of Radiation Oncology, Sunnybrook Health Sciences Centre, Toronto, Canada; ^3^Department of Medical Biophysics, University of Toronto, Toronto, Canada; ^4^Department of Radiation Oncology, University of Toronto, Toronto, Canada; ^5^Department of Surgery, Sunnybrook Health Sciences Centre, Toronto, Canada; ^6^Department of Surgery, University of Toronto, Toronto, Canada; ^7^Department of Medical Imaging, Sunnybrook Health Sciences Centre, Toronto, Canada; ^8^Department of Medical Imaging, University of Toronto, Toronto, Canada; ^9^Department of Electrical and Computer Engineering, York University, North York, Canada; ^10^Division of Medical Oncology, Department of Medicine, Sunnybrook Health Sciences Centre, Toronto, Canada; ^11^Department of Medicine, University of Toronto, Toronto, Canada; ^12^Department of Physics, Ryerson University, Toronto, Canada

**Keywords:** radiomics, MRI, breast cancer, neoadjuvant chemotherapy, biomarkers

## Abstract

Background: Radiomics involving quantitative analysis of imaging has shown promises in oncology to serve as non-invasive biomarkers. We investigated whether pre-treatment T2-weighted magnetic resonance imaging (MRI) can be used to predict response to neoadjuvant chemotherapy (NAC) in breast cancer.

Materials and Methods: MRI scans were obtained for 102 patients with locally advanced breast cancer (LABC). All patients were treated with standard regimens of NAC as decided by the treating oncologist, followed by surgery and adjuvant treatment according to standard institutional practice. The primary tumor was segmented, and 11 texture features were extracted using the grey-level co-occurrence matrices analysis of the T2W-images from tumor cores and margins. Response assessment was done using clinical-pathological responses with patients classified into binary groups: responders and non-responders. Machine learning classifiers were used to develop a radiomics model, and a leave-one-out cross-validation technique was used to assess the performance.

Results: 7 features were significantly (*p* < 0.05) different between the two response groups. The best classification accuracy was obtained using a k-nearest neighbor (kNN) model with sensitivity, specificity, accuracy, and area under curve of 63, 93, 87, and 0.78, respectively.

Conclusions: Pre-treatment T2-weighted MRI texture features can predict NAC response with reasonable accuracy.

## INTRODUCTION

Locally advanced breast cancer (LABC) is defined as breast cancer with tumors greater than 5 cm, primary disease involving the chest wall, skin, or advanced regional lymph node metastasis, without distant metastases [1]. Neoadjuvant chemotherapy (NAC) is the standard of care for patients with LABC, with chemotherapy administered before surgery [2], with the purpose of improving resectability and facilitating breast-conserving therapy [1]. Approximately 50–70% of patients respond to NAC, which can serve as an important surrogate for clinical outcomes in specific molecular subgroups [3]. Pathological response rates are worse, with approximately 20–40% of patients achieving pathological complete response [4]. NAC can lead to various toxicities, including alopecia, fatigue, nausea, myelosuppression, neuropathy, and cardiotoxicity [5]. It would be helpful to develop imaging biomarkers to identify patients likely to benefit from NAC avoiding ineffective treatments in a selected cohort of patients having chemoresistant disease. Radiomic analysis involving a wide range of imaging modalities, including magnetic resonance imaging (MRI) [6–8], quantitative ultrasound (QUS) [9–12], and computed tomography (CT) [13], has shown promising results in the assessment of clinical outcomes for patients with breast cancer.

“Pre” and “post”-treatment MRI scans are often undertaken as the standard of care for patients with LABC for disease staging, assessment of response post-NAC, and surgical decision making. T1 and T2-weighted contrast-enhanced sequences are used to assess tumor extent and characterize malignancy based on morphological characteristics [14]. T2-weighted MRI sequences are based on T2 relaxation time, which is typically dependent on intrinsic tissue characteristics. Factors of the tissue that affect T2 relaxation time include: tissue water and fat content, the random movement of water and macro-molecules, and pH value, with the tissue water content being one of the most influential [15]. Breast cancer lesion T2 relaxation time has shown a decrease after NAC, with a more significant reduction in responders than non-responders [16]. In T2-weighted breast MRI, the texture of these images has been significantly correlated with pathological heterogeneity such that the texture could hypothetically be used to predict patient prognosis. Texture, in that case, was represented in a limited manner by uniformity and entropy, which describe the distributions of intensity histograms of MRI [17]. Also, contrast agents, such as gadolinium, and examining the T1-weighted increase of the tumor intensity over time, one can generate quantitative kinetic texture features representing tumor vascularity characteristics. These features have been demonstrated to have the potential for predicting NAC response before treatment with an area under curve (AUC) of 0.68 [18]. However, not all patients can receive contrast agents due to allergies or renal insufficiencies, and there can be issues with standardizing contrast perfusions and time requirements [19]. Thus, texture features derived from non-contrast T2-weighted MRI are potentially more generalizable, should they show similar predictive capability.

A common and useful method of describing texture involves computing features from the grey-level co-occurrence matrices (GLCMs) of images [20]. GLCMs represent the probability distributions of the pixel intensities between pixels and their neighbors, applied throughout the whole image. Machine learning algorithms, such as Fisher’s linear discriminant analysis (FLD), support vector machines (SVM), and k-nearest neighbors (kNN), permit the tuning of functions or decision boundaries such that classification accuracy is maximized and capable of leveraging the differentiating capacity of GLCM textures [9]. They take advantage of otherwise undetectable multi-dimensional feature relationships.

The study examined T2 non-contrast images in predicting the treatment response to NAC. Specifically, we investigated a number of parameters, including quantization level, region of interest (ROI) selection method (tumor core and margin), GLCM pixel distances, classifier type, and tuning parameters to examine whether treatment response can be predicted before starting NAC.

## RESULTS

The study included 102 patients with LABC treated with NAC. Clinical and pathological details of the analyzed patient cohort are summarized in Table 1. The median age of the patients was 51 years (range: 27–83 years), with the median tumor dimension of 5.2 cm (range: 1.3–12.8 cm). 52% of the patients were premenopausal, 2% were perimenopausal, 40% were postmenopausal, while in the remaining 6% information was not available. In terms of histology, 87% of patients had invasive ductal carcinoma, 6% had invasive lobular carcinoma, mixed invasive ductal and lobular carcinoma in 6%, and invasive micropapillary carcinoma in 1%. Fifty-one percent of patients received doxorubicin and cyclophosphamide followed by paclitaxel (AC-T), 41% received 5-fluorouracil, epirubicin, cyclophosphamide, and docetaxel (FEC-D), and the remaining 8% received other treatments. Trastuzumab was given to 34% of patients. 81% (*n* = 83) of patients were classified as responders, and 19% (*n* = 19) of patients were classified as non-responders.

**Table 1:** Patient, disease, and treatment characteristics (*n* = 102)

**Features**	**Median ± Standard Deviation/Percentage**
**Age**	51 ± 11 years
**Initial Tumour Size**	5.2 ± 2.8 cm
**Histology**	
	Invasive Ductal Carcinoma: 87%
	Invasive Lobular Carcinoma: 6%
	Other: 7%
**Tumour Grade**	
	Grade 1: 7%
	Grade 2: 43%
	Grade 3: 46%
	Unavailable: 4%
**Molecular Features**	
	HR+/HER2-: 41%
	HR+/HER2+: 20%
	HR-/HER2+: 14%
	Triple-negative: 25%
**Neoadjuvant chemotherapy**	
	AC-T (51%)
	FEC-D (41%)
	Others (8%)
**Residual Tumour Size**	1.9 ± 4.1 cm

Abbreviations: HR: Hormone receptor; HER2: Human epidermal growth factor receptor 2; AC-T: doxorubicin and cyclophosphamide followed by paclitaxel; FEC-D: 5-fluorouracil, epirubicin, cyclophosphamide, and docetaxel.

**Figure 1:**
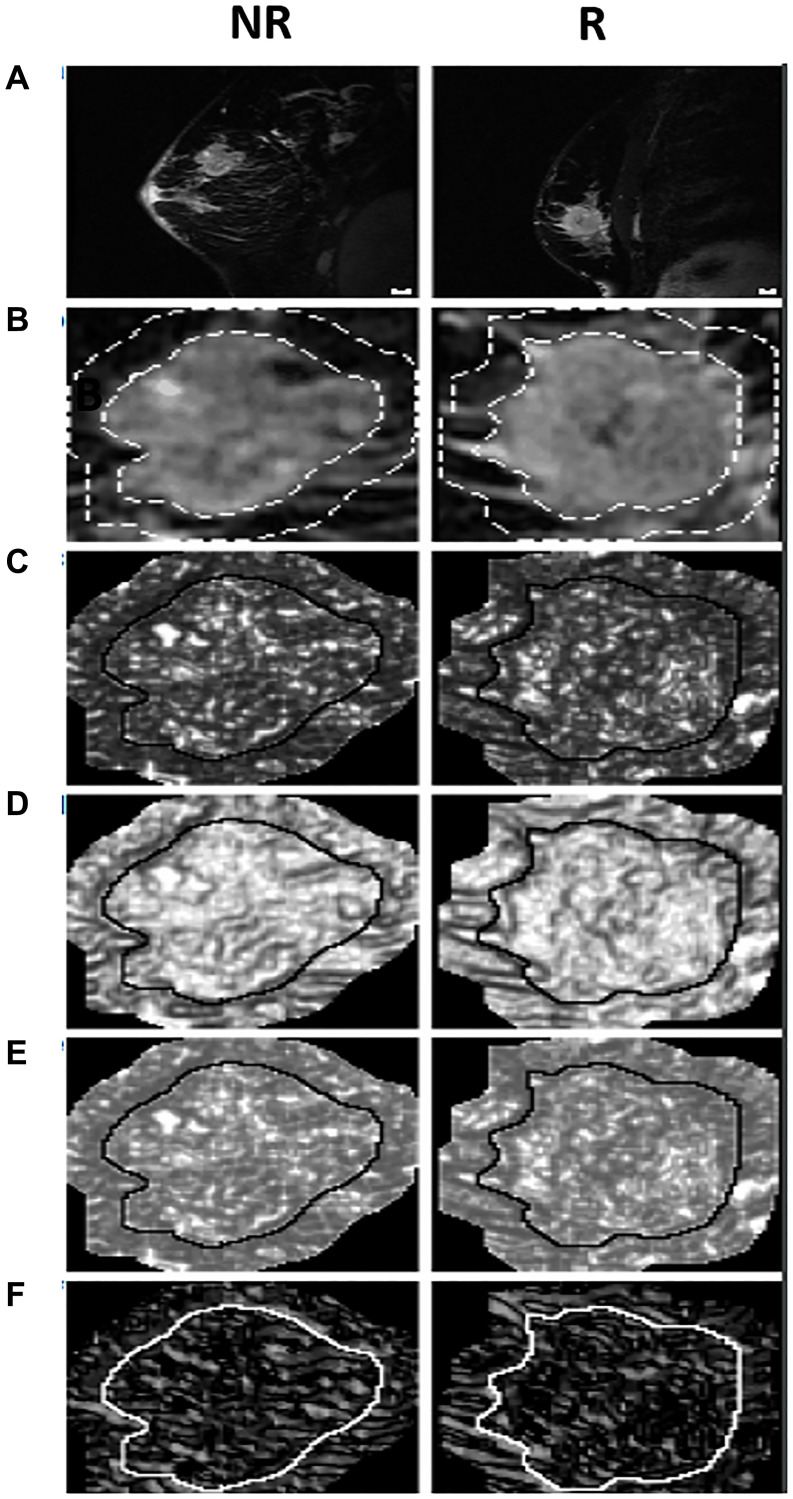
The MRI, quantized tumor ROI, and texture feature images for a typical NR and R. Includes (**A**) MRI sagittal view before treatment, (**B**) the MRI ROI quantized to 16 grey levels with both the ROI core and a margin of 10 pixels outlined, and feature images for (**C**) MAX, (**D**) HOM, (**E**) ENE, and (**F**) COR. The solid lines around the tumor core in the feature images differentiate the cores from the margins. Scale bars on the top panel correspond to 1 cm.

Figure 1 displays representative MRI (A, B) and texture feature images (C–F) for a typical non-responder (NR) and responder (R). For the tumor core, in order of increasing *p*-values, the different texture features were as follows: maximum (MAX), correlation (COR), angular second moment (ASM), energy (ENE), entropy (ENT), standard deviation (STD), variance (VAR), mean (MEA), contrast (CON), homogeneity (HOM), and dissimilarity (DIS).

**Figure 2:**
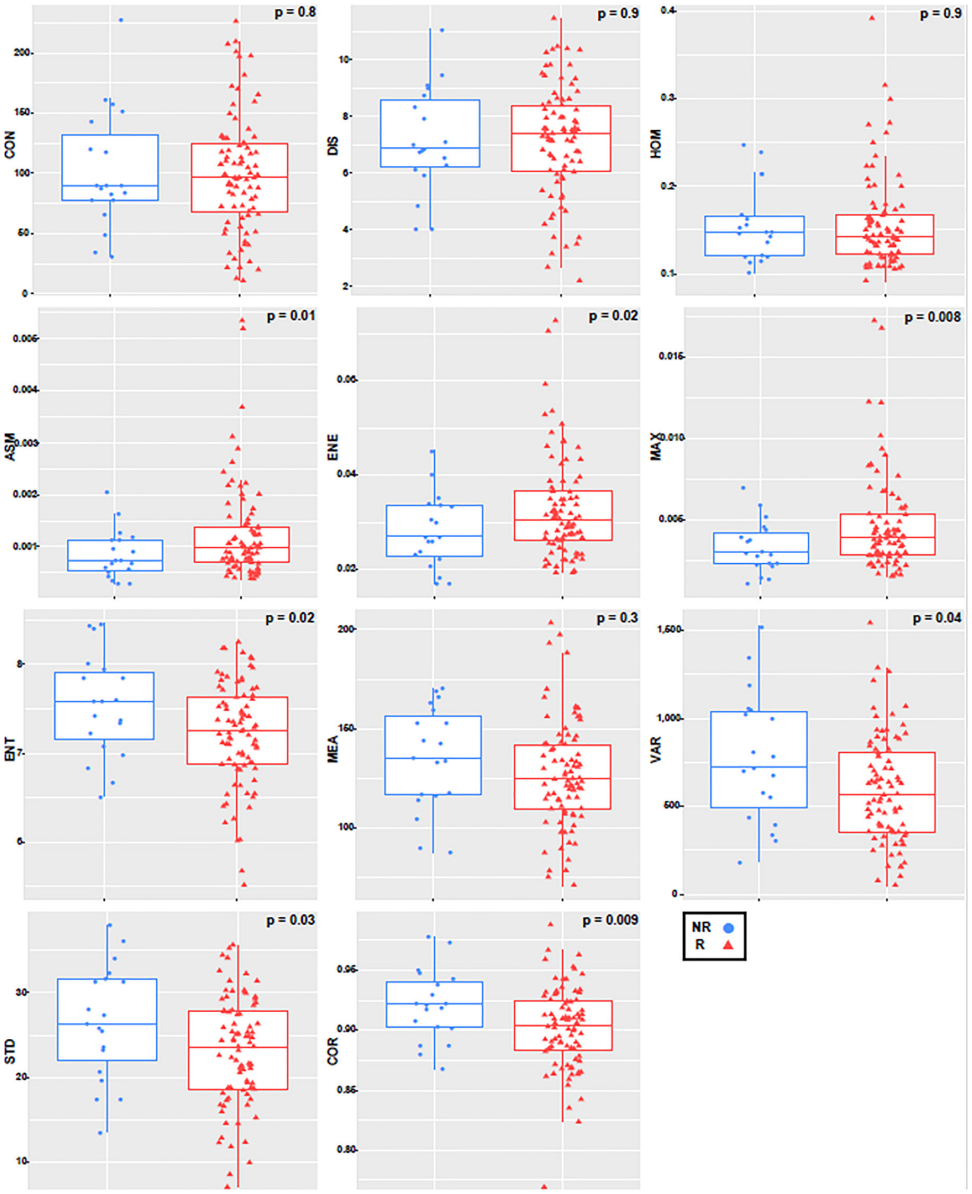
Boxplot with an overlayed scatterplot of the distributions of NR and R patient texture feature values calculated with only the tumor core, a quantization of 256 grey levels, and a pixel distance of 1.

**Figure 3:**
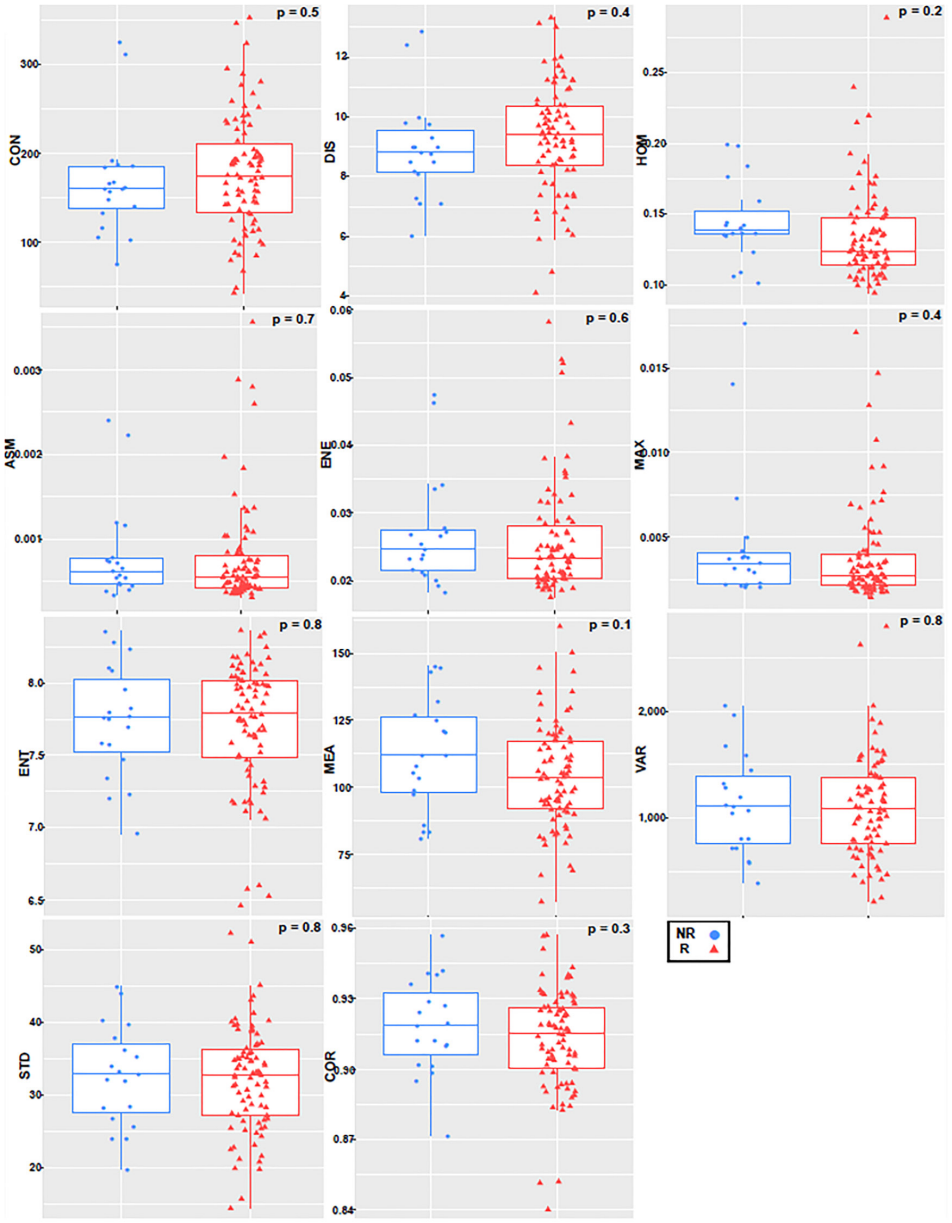
Boxplot with an overlayed scatterplot of the distributions of NR and R patient texture feature values calculated with only tumor margin, a quantization of 256 grey levels, and a pixel distance of 1.

The first seven texture features showing a significant difference between non-responders (NR) and responders (R) (*p* ≤ 0.05), and the final four not showing significant differences between the two groups (*p* > 0.05). For the tumor margin, none of the features were independently significant. Figures 2 and 3 represent the distributions of the different texture feature values for NR and R obtained from tumor core and margin, respectively.

**Figure 4:**
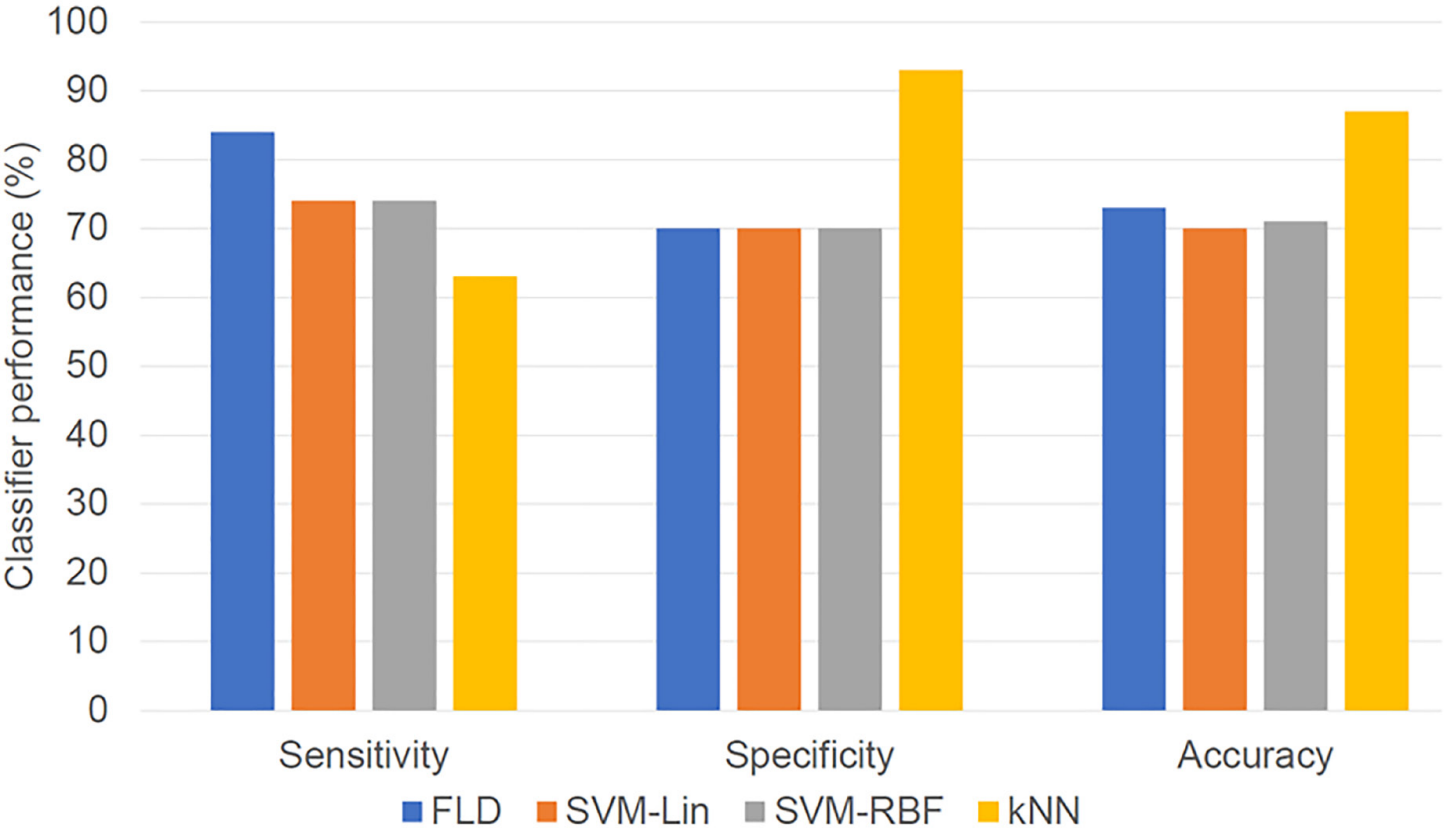
Bar diagram showing classifier result values (optimized for maximum F1-Score). The performances of the four classifiers in terms of sensitivity, specificity, and accuracy are presented.

The classifier performances using the individual machine learning algorithms are summarized in Table 2. The kNN classifier had the maximum accuracy (87%) and specificity (93%) in the overall analyses, but the FLD classifier had the maximum sensitivity (84%) and F1-Score (0.76). Both optimal SVM classifiers used the ROI core texture features, whereas the FLD and kNN used the texture features from the margin, including 15 and 10 pixels, respectively. Figure 4 shows the bar diagram comparing the different classifiers (FLD, SVM-Lin, SVM-RBF, kNN) with sensitivity, specificity, and accuracy as performance evaluation metrics.

**Table 2:** Optimal patient response classifier parameter details, including texture features used

Classifier	Sensitivity (%)	Specificity (%)	Accuarcy (%)	AUC	F1-Score	Features	ROI Selection Method	Quantization	Pixel distance
**FLD**	84	70	73	0.74	0.76	MEA, VAR, STD	Margin (15 pixels)	128	1
**SVM-Lin**	74	70	70	0.75	0.72	MEA, STD	Core	32	5
**SVM-RBF**	74	70	71	0.76	0.72	MEA, STD, ENT, MAX	Core	32	5
**kNN**	63	93	87	0.78	0.75	HOM, ASM	Margin (10 pixels)	128	1

Abbreviations: AUC: Area under curve; ROI: Region of interest; MEA: Mean; VAR: Variance; STD: Standard deviation; ENT: Entropy: MAX: Maximum; HOM: Homogeneity; ASM: Angular second moment.

**Figure 5:**
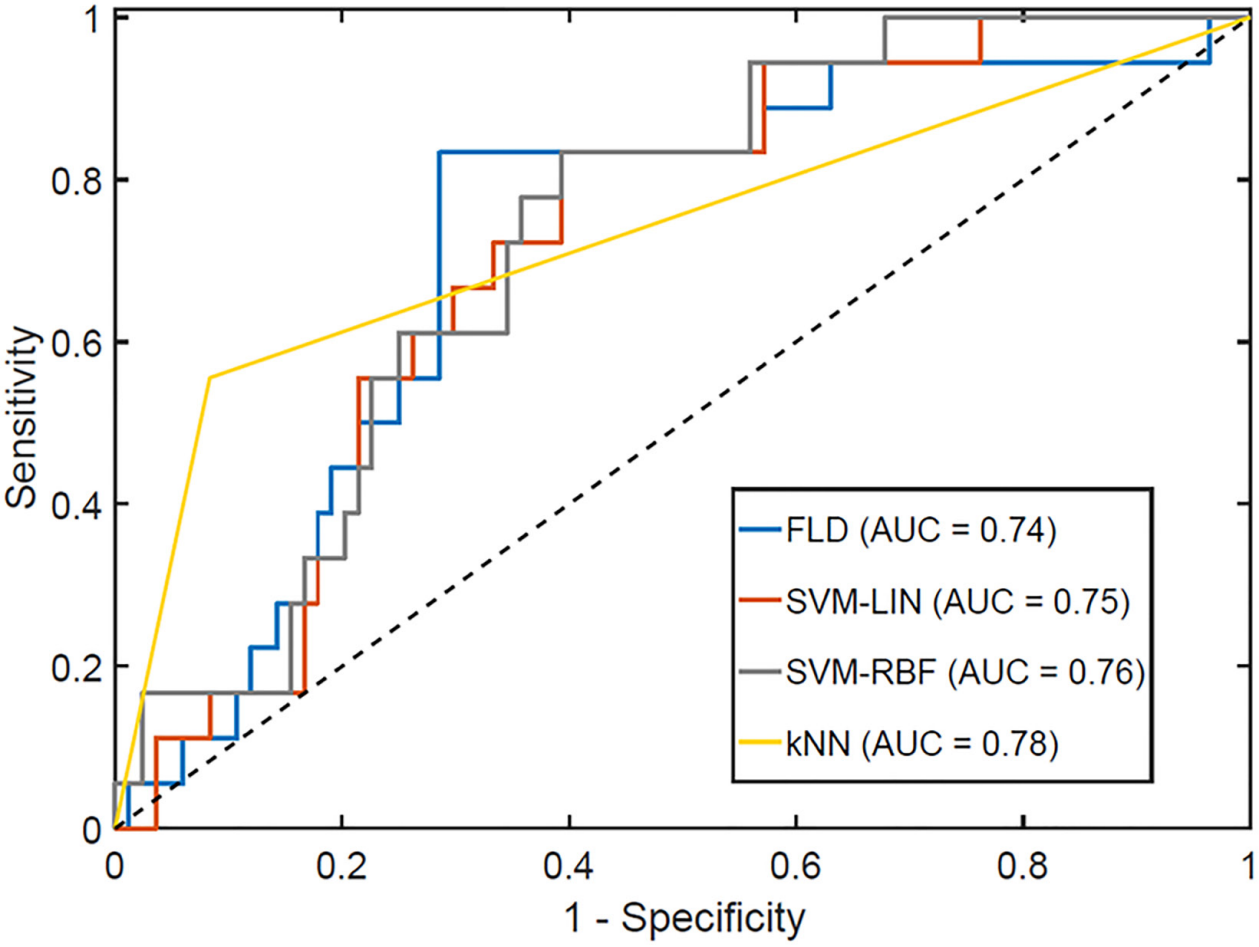
Receiver operator characteristics curve for the optimal response classifiers, optimized for maximum F1-Score. The AUCs for each classifier are reported in the legend.

Full results associated with different quantizations, pixel distances, and tumor core and margin analyses are presented in Supplementary Figure 1. For the quantization, the optimal value depended on classifier type, with the highest accuracy associated with a classifier using 256 grey levels for FLD, 32 grey levels for SVM-Lin and SVM-RBF, and 16 grey levels for kNN, with a fixed ROI selection method of the core and a pixel distance of 1 (Supplementary Figure 1A). When varying pixel distance between 1 and 15, there was no significant change in classification performance (Supplementary Figure 1B), whereas the results for 32 vs. 128 grey levels had different trends (Supplementary Figure 1C). For the ROI selection method, a margin of 15 pixels was consistently the least sensitive with quantization of 32 grey levels but consistently the most sensitive with quantization of 128 grey levels. (Supplementary Figure 1D and 1E) Additionally, 128 grey levels resulted in higher accuracies than 32 grey levels for all classifiers except SVM-Lin. Across all models trained using 128 grey levels, those trained using the margins had better accuracy than those from the core. A quantization of 128 grey levels with the margin of 10 pixels had the individual classifier with the highest overall accuracy (kNN, Acc = 87%). Models were tested using the core and margin features together. This did not improve performance, as the features selected in the optimal model were solely from the margin. This was a significantly better performance than from tumor core features (accuracy 87% versus 71%). Figure 5 presents the receiver operator characteristics curves for each of the optimal classifiers and displays their area under curve (AUC) values. The FLD classifier had the minimal AUC, with 0.74, SVM-Lin had 0.75, SVM-RBF had 0.76, and kNN exhibited an AUC of 0.78.

## DISCUSSION

Radiomics is an emerging discipline in medicine and oncology involved with the advanced processing of imaging data [21]. Radiomic analysis can be undertaken on various morphological or functional imaging modalities undertaken in routine management for patients with cancers. The various features extracted can include first-order features including shape parameters, second-order features like texture, or higher-order, which can extract meaningful information which is otherwise beyond the abilities of human interpretation. Computational imaging analysis leads to generation of higher-dimensional data, which is often coupled with advanced machine learning classifiers for meaningful interpretation of the data towards specific biological endpoints. Supervised machine learning algorithms are more commonly used for development of radiomic models using the extracted set of imaging features directed towards a pre-specified output. Quantitative imaging can therefore serve as crucial non-invasive biomarkers linked to clinical outcomes like treatment response, survival, or determination of treatment-related toxicities, guiding treatment decisions in clinical practice. Radiomics research has been widely undertaken in breast cancer using different imaging modalities, including CT, MRI, and QUS [22]. In this study, we explored the efficacy of pre-treatment non-contrast T2-weighted MRI in predicting response to NAC in a cohort of 102 patients with LABC, incorporating different regions of the tumor (core vs. margin) and different techniques of data processing (quantization, pixel distance).

Similar work carried out using T2-weighted MRI texture has either analyzed different endpoints [6], not examined solely pre-treatment predictive capacity [7], not examined T2-weighted MRI independently [23], or been incapable of detecting a significant difference [24, 25]. Henderson et al. used GLCM entropy with a pixel distance of 2 and 5, and a constant quantization of 64 grey levels [24], involving 2 features, and did not take advantage of any ML techniques with data from 88 patients. Additionally, the analysis that was performed was on T2-weighted MRI without fat suppression [24]. Additional work by Liu et al. was more comprehensive, with a cohort of 414 patients using a total of 13,950 radiomic features per patient, including 90 textural features [25]. It used advanced feature selection, fat-suppressed pre-treatment T2-weighted MRI, and a final SVM-RBF model with 10-fold cross-validation. Their model constructed with only T2-weighted MRI had AUC = 0.69, *p* = 0.042, predicting pathologic complete response [25].

In this study, the ROI selection method was varied to examine the effects of the tumor core alone and tumor margins of different sizes. Quantitative ultrasound research on NAC breast cancer response has demonstrated that the tumor boundary contents can be significant in predicting patient response [9]. Independent of analysis and classifier (except for the 32 grey-level SVM), the classifiers trained with the pixels from the border with either 5, 10, or 15 pixels outperformed the classifiers trained on the tumor core alone. The peritumoral region can represent areas of microscopic tumor as well as the tumor-infiltrating lymphocytes playing an essential role in dictating response to chemotherapy.

Our working model is that as tumors become more aggressive and less likely to respond to chemotherapy, their structure becomes more disorganized. This is in alignment with the fact that as grade increases, tumors become less like normal breast tissue and more aggressive. Whereas pathologists limit characterization to 3 tumor grades, recent genetic tests have been better able to categorize tumor subsets based on genetic “disorganization”. Here we postulate that this characterization is based rather on structural disorganization with indirect links to the genetic alterations responsible for changes in cellular structure and higher-order organization that changes with the development of malignant neoplasia.

One limitation of the current study was the use of different chemotherapy regimens. While a model that is invariant of treatment type is useful, a variety of models trained only for a specific treatment type might in the future yield a greater classification accuracy. Additional texture features could be extracted from tumors using alternative methods of representation such as grey run-length matrices (GRLMs), grey-level size zone matrices (GLSZMs), and first-order features, which will be included in future follow up studies. We intend to expand the current study cohort to include a higher number of patients to perform more robust validation strategies, including consideration of external validation from a different institution. In comparison to other studies, we had used a different endpoint towards detection of chemotherapy response. Rather than using pathological complete response as an endpoint, we had used responder and non-responder classification outputs. We believe in clinical practice; it will be more prudent to continue NAC in patients demonstrating some or partial response rather than considering a stricter criterion of complete response.

## MATERIALS AND METHODS

### Patient selection and treatment information

This study was approved by the research ethics board of Sunnybrook Health Sciences Centre, Toronto. Patients with LABC who received NAC were included in this retrospective study. Waiver of consent was approved by the ethics committee, given the retrospective nature of the study.

After NAC, surgery was performed, followed by radiation therapy, according to institutional guidelines. Before treatment initiation, a core needle biopsy was acquired from each patient for histopathological confirmation and assessment of molecular characteristics. MRI images were obtained before starting NAC.

The response was classified using a modified RECIST score, using a combination of post-NAC imaging and histopathological specimen, which served as the gold standard [26]. Patients were classified into binary treatment response groups: responders and non-responders. Patients with a complete pathological response, residual tumor cellularity of less than 1%, and/ or decrease in tumor dimension by more than 30%, were classified as “responders”. Patients with partial response not defined by the previous criteria or having progressive disease were labeled “non-responders”.

### MRI protocol

All patients were scanned with a 1.5 T Signa HDxt (GE Medical Systems) with 8 breast-specific receiving coils. The sequences analyzed were from sagittal T2-weighted scans pre-contrast (fast spin echo, fat-saturated, TR 2500 ms, variable TE averaging ~76.14 ms, 90-degree flip angle, slice thickness 3 mm, with a variable DFOV, averaging approximately 190 mm). All MRI data were obtained from the same scanner dedicated to breast imaging at a single institution.

### Image analysis segmentation

The ROI was selected freehand using the lasso tool from GIMP (GIMP 2.10) guided by a radiologist specializing in breast radiology. The ROIs were selected on a per-slice basis, spanning the tumor volume on the sagittal T2 pre-treatment MRI. Also, for tumor margin, a set of additional ROIs was created, which expanded on the original ROI by 5, 10, and 15 pixels in all directions within the breast tissue.

### Texture features

Grey-level co-occurrence matrices (GLCMs) represent pixel contrast probabilities determined from an image and were used to calculate Haralick’s texture features [27, 28]. All texture feature values used to train the classifiers in this study were directionally invariant. GLCM offset direction was kept fixed, and offset distance was varied to capture difference patterns. Whereas for most MRI image analyses, a pixel distance of 1 is selected [29, 30], the original ROI was tested with offset distances of 1, 2, 3, 4, 5, 10, and 15 pixels. For the ROI-margin analysis, the only offset distance used was 1 pixel, as there were not enough pixels to get significant results with larger pixel distances.

Quantization or grey-level binning was performed after the ROI had been selected, so any data from outside of the tumor mass did not influence the process. Quantization was varied for the purpose of this work, with values of 16, 32, 64, 128, and 256 grey levels, since in the literature, there was no consensus on the optimal value for MRI analysis [29, 31].

A set of 11 features including contrast (CON), dissimilarity (DIS), homogeneity (HOM), angular second moment (ASM), energy (ENE), maximum (MAX), entropy (ENT), mean (MEA), variance (VAR), standard deviation (STD), and correlation (COR) were determined for the T2-weighted ROIs. These features were described and compiled by Hall-Beyer [27]. Feature extraction was done using a custom Python script (code can be made available upon request). The features were extracted individually from the tumor core and margin.

### Data classification

For each of the machine learning methods below, the data were tested using 1–4 features for each parameter combination. Limiting the maximum number of features to 4 was done to avoid the curse of dimensionality [32]. Machine learning was done using MATLAB (R2016a).

Algorithms investigated maximized the accuracy in the first execution and the F-score in the next. Algorithms returned the features used in the generation of the classifier in addition to the values of all tuning parameters (e.g., *k* values for kNN). The algorithm assessed the sensitivity, specificity, accuracy, F-score, and AUC as evaluation metrics. In this study, we defined true positives as non-responders. The results were tested with leave-one-out cross-validation. This method regenerated the model using all the available data but withholding data from a single patient, running such that all patients are left out in one instance and generating an average of the classifier performance evaluation metrics.

The first data classification machine learning technique used was Fisher’s linear discriminant (FLD) analysis [33]. This was tested with both balanced and unbalanced data sets. The balanced data set was generated by subsampling the original data into several subsets, such that each subset had an equal number of R and NR. In this case, the balanced subset contained 19 R and 19 NR. FLD analysis created an axis that had a maximized separability between the two classes-responders (R) and non-responders (NR).

Additionally, support vector machines (SVMs) were used, with two kernel functions: linear (Lin) and radial basis function (RBF). SVMs use the kernel function to find a hyperplane to differentiate the data. The SVMs were executed on a balanced dataset. Within SVM analysis, two classifier parameters were tuned so that the model better fit the data when using the RBF as the kernel. These include the C and the γ parameters (C representing the cost of misclassification, and γ controlling the shape of the kernel function). The optimal C and γ were selected by grid search in the ranges of C = 2^1^ to 2^10^ and γ = 2^−15^ to 2^5^.

The final algorithm investigated was k-nearest neighbors (kNN), a technique that classifies the data depending on the classes of the k nearest neighbors (where *k* is variable). *k* was varied from 1–5 neighbors with an increment of 1.

The best overall classifier was determined from identical image datasets with different imposed grayscale quantization levels. Classifiers also evaluated texture features from the tumor core and margin, in addition to different GLCM pixel distances.

### Ethics approval and consent

The study was conducted following the Declaration of Helsinki. The ethics committee of Sunnybrook Health Sciences Centre, Toronto, was involved in study approval (REB: 034-2020), necessary data monitoring, and appropriate conduct of the research. The ethics committee approved the waiver of consent given the retrospective nature of the study.

## CONCLUSIONS

In conclusion, classifiers trained with pre-treatment MRI texture were shown to have an accuracy of 87% in predicting NAC treatment response. Here a classifier based on tumor margin performed better than one based on tumor core alone. Radiomic analysis of MR images using simple machine learning classifiers could predict response and guide clinicians in prescribing effective treatments.

## SUPPLEMENTARY MATERIALS



## Abbreviations

MRI: Magnetic resonance imaging
NAC: Neoadjuvant chemotherapy
LABC: Locally advanced breast cancer
QUS: Quantitative ultrasound
CT: Computed tomography
AUC: Area under curve
GLCM: Grey-level-co-occurrence matrix
FLD: Fisher’s Linear discriminant
SVM: Support vector machine
kNN: *k*-nearest neighbor
ROI: Region of interest
AC-T: Doxorubicin, Cyclophosphamide, Paclitaxel
FEC-D: 5-Fluorouracil, Epirubicin, Cyclophosphamide, Docetaxel
MAX: Maximum
COR: Correlation
ASM: Angular second moment: ENE: Energy
ENT: Entropy
STD: Standard deviation
VAR: Variance
MEA: Mean
CON: Contrast
HOM: Homogeneity
DIS: Dissimilarity (DIS)
NR: Non-responder
R: Responder
GRLM: Grey run-length matrix
GLSZM: Grey-level size zone matrix
