# Supplementary material for: MRI texture features from tumor core and margin in the prediction of response to neoadjuvant chemotherapy in patients with locally advanced breast cancer
Source: Oncotarget. 2021 Jul 6;12(14):1354–65. doi: 10.18632/oncotarget.28002 (PMC8274727; doi:10.18632/oncotarget.28002)
Supplement: Supplementary file 1 [file oncotarget-12-1354-s001.pdf]

MRI texture features from tumor core and margin in the prediction of response to neoadjuvant chemotherapy in patients with locally advanced breast cancer

SUPPLEMENTARY MATERIALS

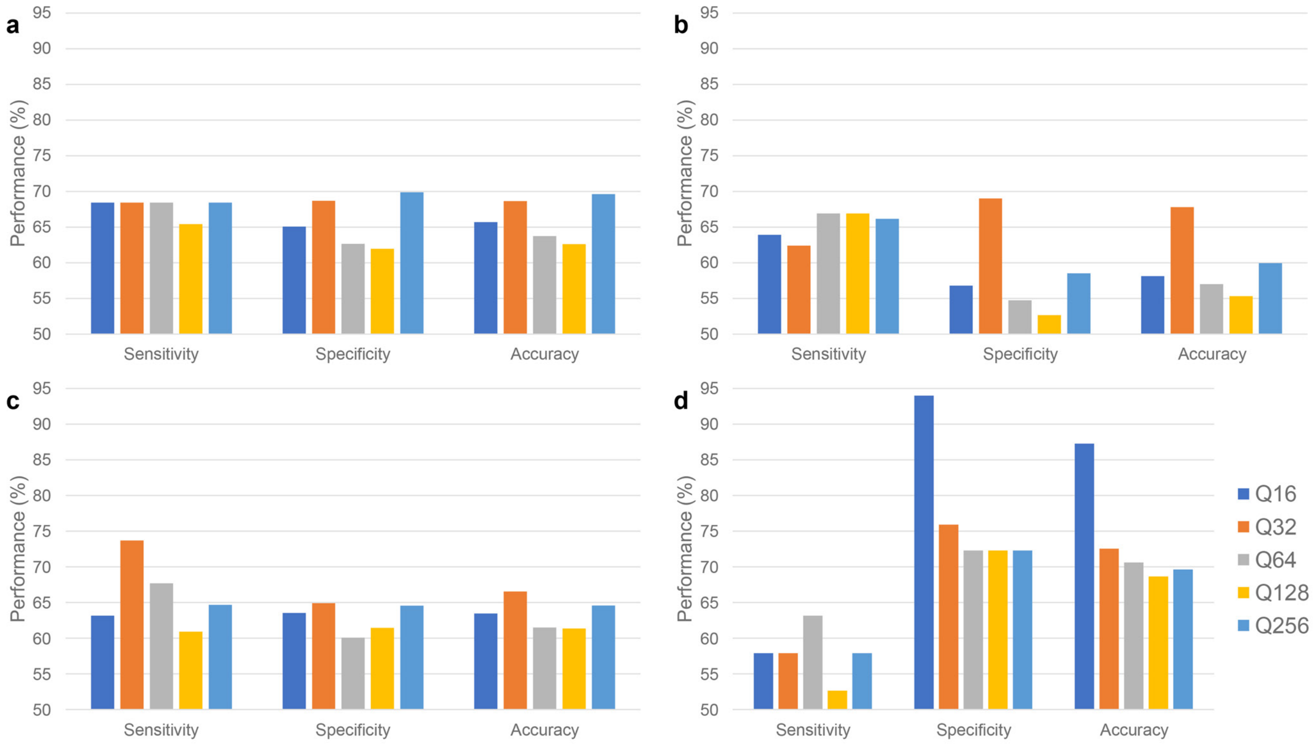

Supplementary Figure 1A: Classification performance for FLD (**a**), SVM-Lin (**b**), SVM-RBF (**c**), and kNN (**d**) with varying quantization. Each bar class represents a different maximum number of grey levels. The optimal classifier was defined as the classifier with the maximum F1-Score. The border selection method used the ROI core, and the pixel distance was fixed at 1 pixel.

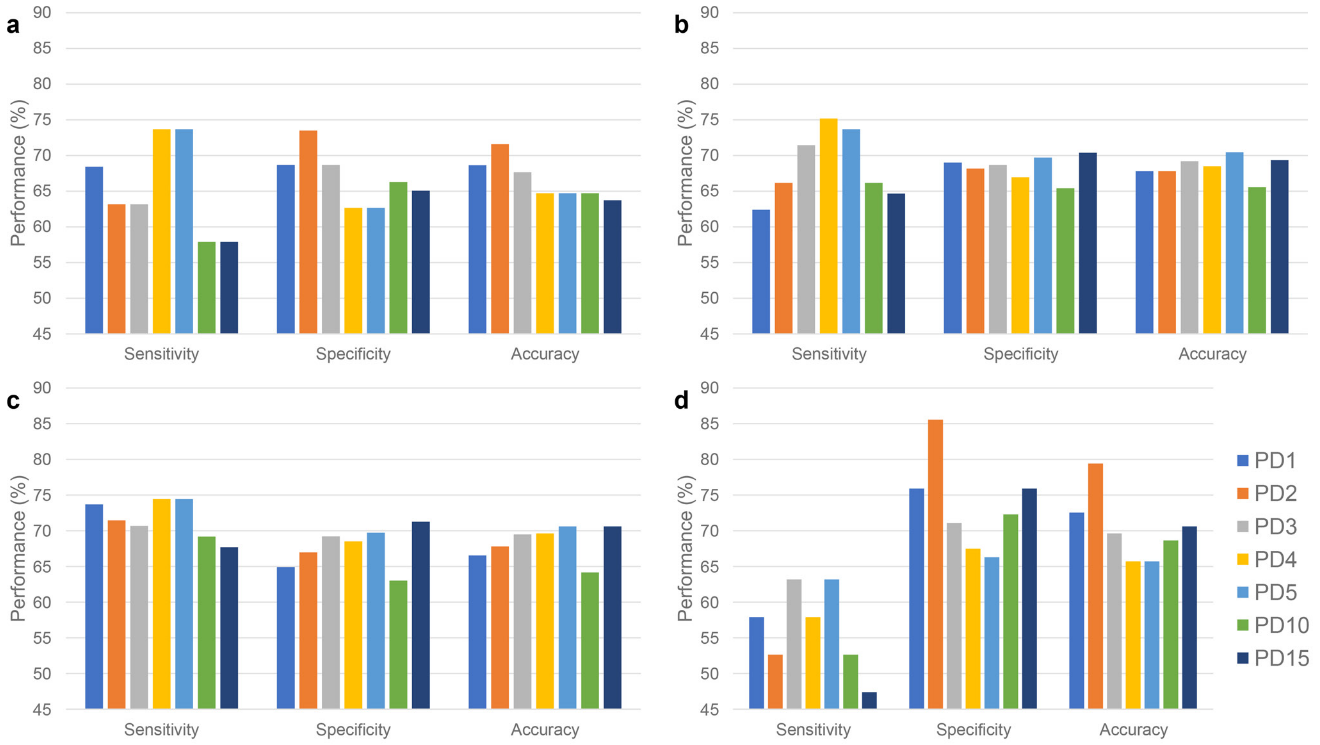

Supplementary Figure 1B: Classification performance for FLD (**a**), SVM-Lin (**b**), SVM-RBF (**c**), and kNN (**d**) with a varying pixel distance. Each bar class represents a different pixel distance in the number of pixels. The optimal classifier was defined as the classifier with the maximum F1-Score. The border selection method used the ROI core, and the quantization was fixed at 32 grey levels.

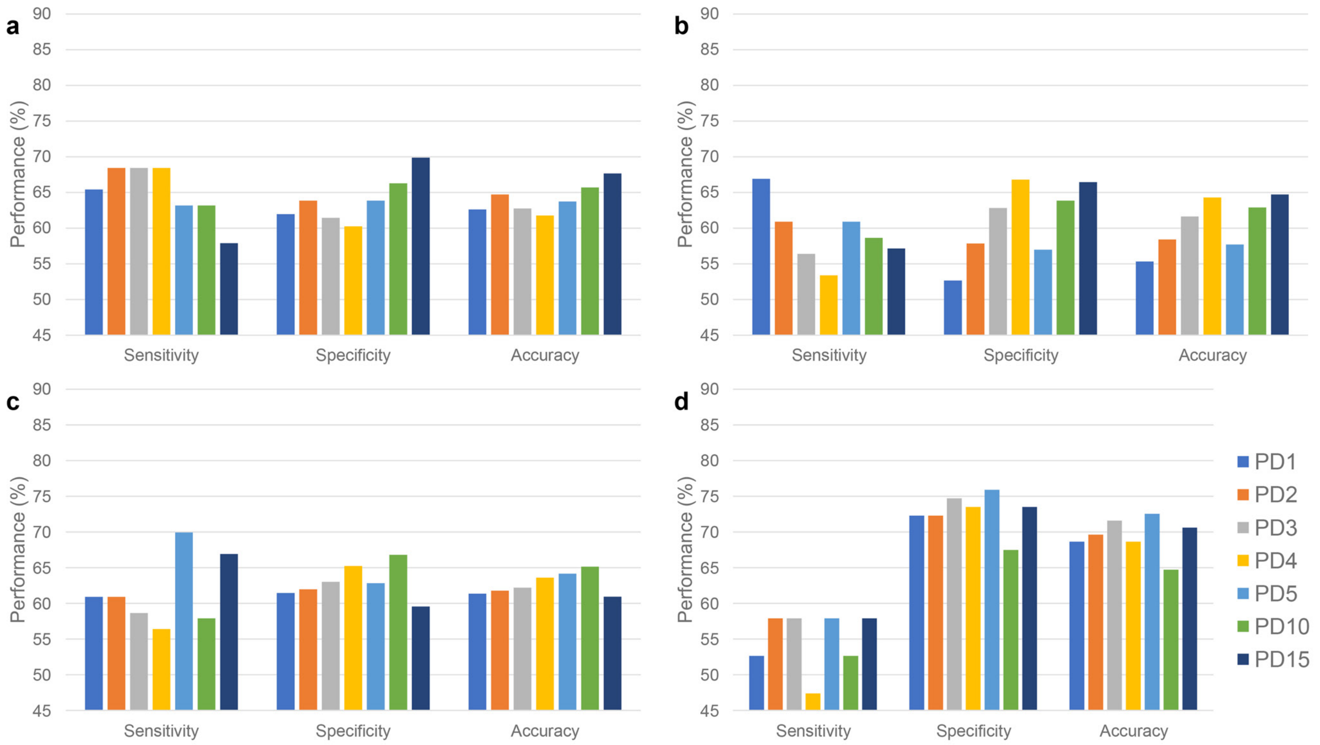

Supplementary Figure 1C: Classification performance for FLD (**a**), SVM-Lin (**b**), SVM-RBF (**c**), and kNN (**d**) with a varying pixel distance. Each bar class represents a different pixel distance in the number of pixels. The optimal classifier was defined as the classifier with the maximum F1-Score. The border selection method used the ROI core, and the quantization was fixed at 128 grey levels.

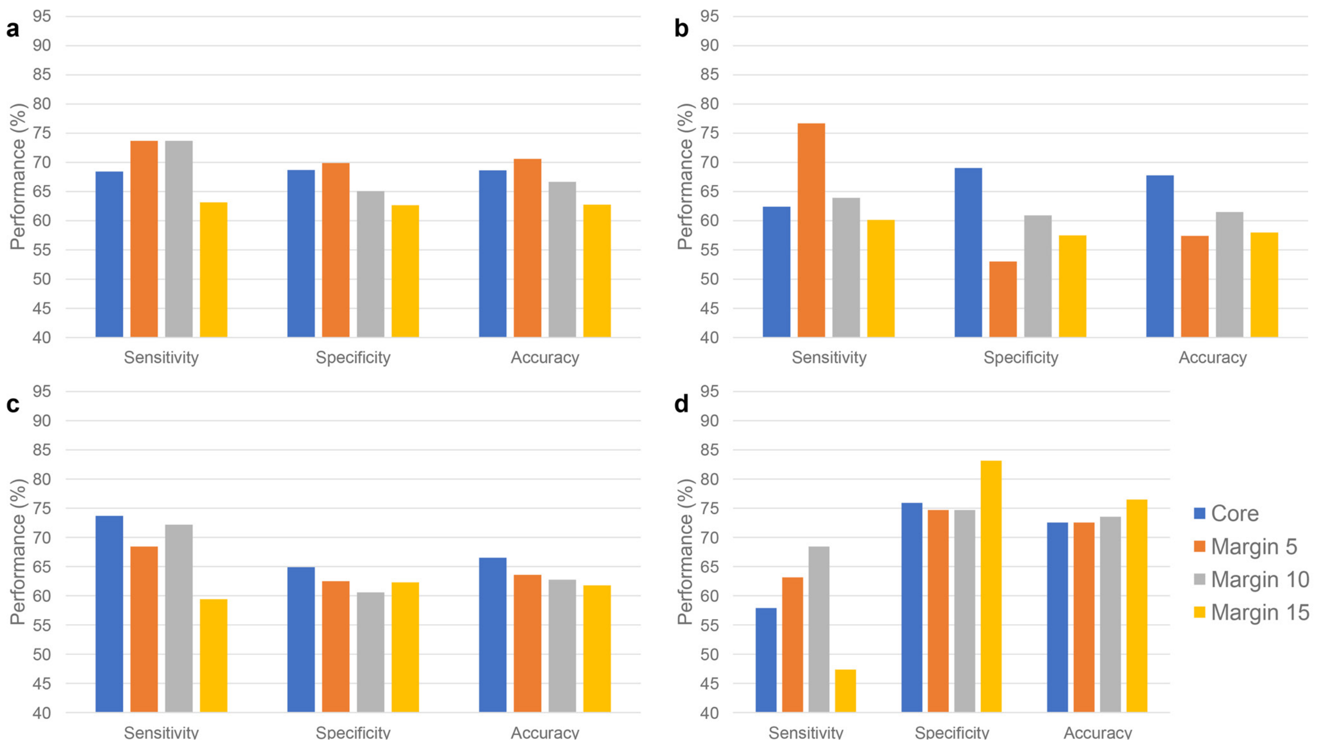

Supplementary Figure 1D: Classification performance for FLD (**a**), SVM-Lin (**b**), SVM-RBF (**c**), and kNN (**d**) with a varying ROI selection method. Each bar class represents the ROI selection method, with Core being the tumor mass and Margin being the tumor margin with a width in pixels. The optimal classifier was defined as the classifier with the maximum F1-Score. The quantization was fixed at 32 grey levels, and the pixel distance was set at 1 pixel.

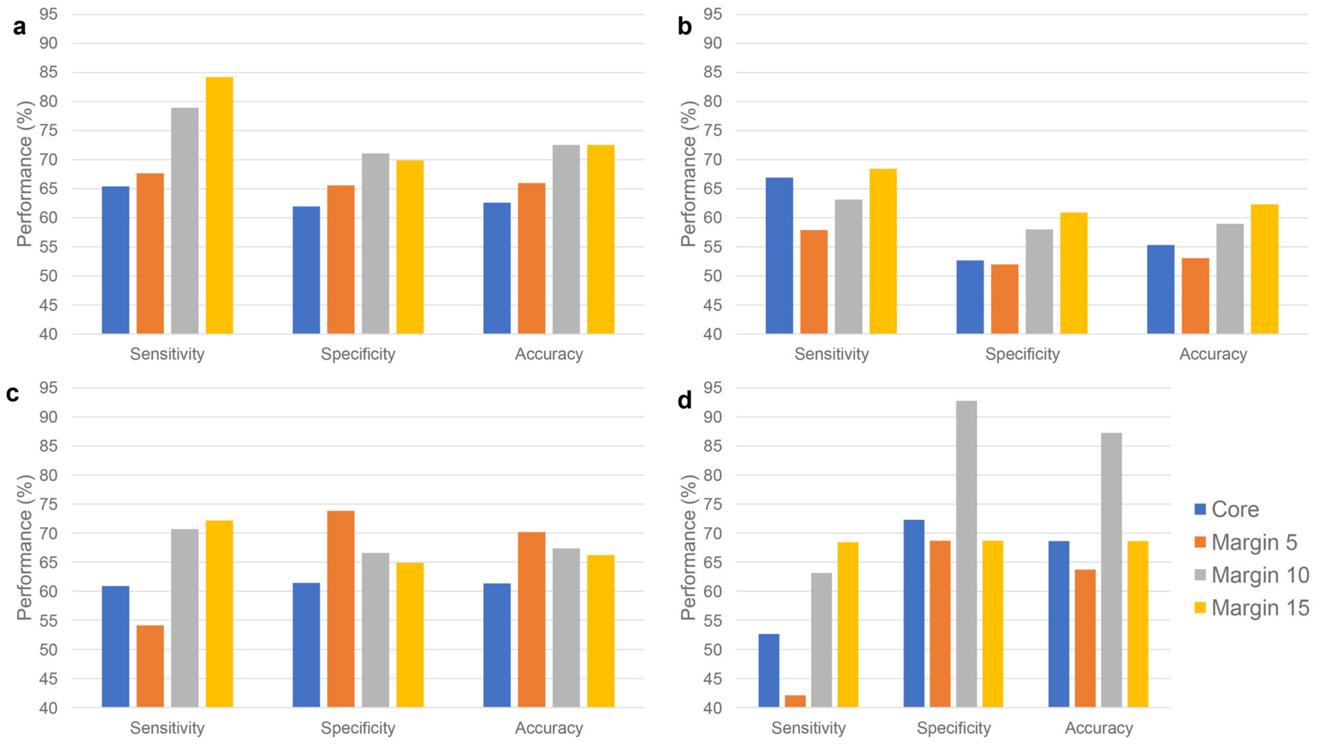

Supplementary Figure 1E: Classification performance for FLD (**a**), SVM-Lin (**b**), SVM-RBF (**c**), and kNN (**d**) with a varying ROI selection method. Each bar class represents the ROI selection method, with Core being the tumor mass and Margin being the tumor margin with a width in pixels. The optimal classifier was defined as the classifier with the maximum F1-Score. The quantization was fixed at 128 grey levels, and the pixel distance was set at 1 pixel.
